# Prevenção, emancipação social e transição paradigmática: uma trajetória interdisciplinar de 40 anos no Brasil sobre acidentes e desastres

**DOI:** 10.1590/0102-311XPT169123

**Published:** 2024-05-20

**Authors:** Marcelo Firpo Porto

**Affiliations:** 1 Escola Nacional de Saúde Pública Sergio Arouca, Fundação Oswaldo Cruz, Rio de Janeiro, Brasil.

**Keywords:** Disaster Prevention, Epistemology, Social Inequality, Empowerment, Prevenção de Desastres, Epistemologia, Iniquidade Social, Empoderamento, Prevención de Desastres, Epistemología, Iniquidad Social, Empoderamiento

## Abstract

O artigo, na forma de ensaio, sistematiza uma trajetória profissional de experiências interdisciplinares e socialmente engajadas em torno da análise e prevenção de acidentes e desastres nos últimos 40 anos. O trabalho acadêmico se desenvolveu principalmente no âmbito da pesquisa e pós-graduação na saúde pública brasileira impulsionado pelo movimento sanitarista e a construção do Sistema Único de Saúde (SUS) em sua busca por democracia, justiça social e sanitária. A base empírica envolveu ações de vigilância em saúde dos trabalhadores e ambiental organizadas em redes protagonizadas pelo SUS em conjunto com universidades, sindicatos, movimentos sociais, organizações não governamentais (ONG) ambientalistas e Ministérios Públicos. Eventos de maior complexidade socioambiental em setores como siderurgia, petroquímico, mineração, agronegócio e energia forjaram a busca por novos referenciais epistêmicos e interdisciplinares que abarcam duas novas justiças: a ambiental e a cognitiva. Este artigo apresenta essa trajetória de contribuições conceituais em três movimentos a partir da década de 1980 até os dias atuais, cada qual correspondendo a um contexto sociopolítico e institucional, para pensar movimentos de transição paradigmática na análise e prevenção de acidentes e desastres numa perspectiva interdisciplinar. Finaliza-se com a sugestão de prevenção abissal e emancipatória para enfrentar diferentes crises da atualidade, como a ambiental, a sanitária, a democrática e a civilizatória.

## Abstract (en)

The article, in the form of an essay, systematizes a 40-year-long professional trajectory of interdisciplinary and socially engaged experiences around the analysis and prevention of accidents and disasters. This study was mainly developed within the scope of research and postgraduate studies in Public Health in Brazil, driven by the sanitarian movement and the construction of Brazilian Unified National Health System (SUS) in its search for democracy and social and health justices. Its empirical basis involved workers’ health and environmental surveillance actions organized in networks led by SUS in conjunction with universities, unions, social movements, environmental nongovernmental organizations (NGO), and Public Prosecutors’ Offices. Events of greater socio-environmental complexity in sectors such as steel, petrochemicals, mining, agribusiness, and energy forged the search for new epistemic and interdisciplinary references that encompassed two new justices, i.e., environmental and cognitive. This essay systematizes this trajectory of conceptual contributions in three movements from the 1980s to the present day (each corresponding to a socio-political and institutional context) to reflect on paradigmatic transition movements in the analysis and prevention of accidents and disasters from an interdisciplinary perspective. It ends by suggesting abyssal and emancipatory prevention to face different current crises, including environmental, health, democratic, and civilizing ones.

## Resumen

El artículo, en forma de ensayo, sistematiza una trayectoria profesional de experiencias interdisciplinarias y socialmente comprometidas en torno al análisis y la prevención de accidentes y desastres en los últimos 40 años. El trabajo académico se desarrolló principalmente en el ámbito de la investigación y postgrado en Salud Colectiva brasileña, impulsado por el movimiento sanitario y la construcción del Sistema Único de Salud (SUS) en su búsqueda por democracia, justicia social y sanitaria. La base empírica involucró acciones de vigilancia en salud y ambiental de los trabajadores, organizadas en redes protagonizadas por el SUS en conjunto con universidades, sindicatos, movimientos sociales, organizaciones no gubernamentales ambientalistas y Ministerios Públicos. Los acontecimientos de mayor complejidad socioambiental en sectores como la siderurgia, el petroquímico, la minería, el agronegocio y la energía han llevado a la búsqueda de nuevas referencias epistémicas e interdisciplinarias que abarcaron dos nuevas formas de justicia, la ambiental y la cognitiva. El artículo sistematiza esa trayectoria de contribuciones conceptuales en tres movimientos a partir de la década de 1980 hasta los días actuales, cada cual, correspondiendo a un contexto sociopolítico e institucional, para pensar movimientos de transición paradigmática en el análisis y prevención de accidentes y desastres desde una perspectiva interdisciplinaria. Se finaliza con la sugerencia de prevención abisal y una prevención emancipadora para enfrentar diferentes crisis de la actualidad, como la ambiental, la sanitaria, la democrática y la de civilización.

## Introdução: transição paradigmática e três movimentos no contexto pós-ditadura militar

Este artigo busca resgatar e sistematizar a apropriação de alguns conceitos e concepções sobre a análise e prevenção de acidentes e desastres, por uma visão interdisciplinar e socialmente engajada, como estratégia epistemológica de transição paradigmática e política de transformação social. Nosso objetivo é inspirar processos emancipatórios dentro da Saúde Coletiva e de outros campos que integram conhecimentos e práticas distintos.

Para isso, elegemos o tema de análise e prevenção de acidentes e desastres para refletir sobre possibilidades de transição paradigmática ocorridos na trajetória interdisciplinar do autor envolvendo diferentes coletivos nas últimas quatro décadas. Ao longo do tempo, foram feitos esforços de incorporação de referenciais e conceitos acionados como estratégia epistemológica para enfrentar desafios e propostas de transformação social em contextos políticos, institucionais e acadêmicos marcados por uma sociedade periférica e subalternizada que buscava se libertar das amarras de uma ditadura militar que impedia avanços por democracia, justiça social e saúde.

Mesclando elementos de ensaio reflexivo e revisão narrativa, este artigo é uma sistematização das experiências acadêmicas, institucionais e engajadas do autor inserido em diferentes coletivos acadêmicos e institucionais, em particular os relacionados ao Sistema Único de Saúde (SUS), ao Ministério Público e a movimentos sociais, como sindicatos e organizações ambientalistas. Ou seja, momentos nos quais se organizavam e atuavam coletivos contra-hegemônicos que lutavam por democracia, saúde, melhores condições de vida e trabalho, e direitos territoriais. Como veremos, a busca por transição paradigmática, foco do artigo, significa mudar concepções e referenciais sobre como os acidentes e os desastres ocorrem, quais suas consequências, suas formas de prevenção e qual o papel dos vários sujeitos sociais, como trabalhadores, sindicatos, organizações não governamentais (ONG) e diversas instituições. Por limitações de tamanho do artigo, não serão detalhados o modus operandi e as características empíricas das experiências de campo que propiciaram criar espaços e aplicar as novas concepções de análise e prevenção de acidentes e desastres na busca por transição paradigmática em cada movimento da trajetória apresentada.

Uma das inspirações para o ensaio provém de Oscar Jara ^1^, educador popular e sociólogo que nos ajuda a pensar de forma criativa na experiência para a construção de conhecimentos. Jara propõe a sistematização de experiências para articular, de forma original e crítica, referenciais teóricos e singularidades como fontes de aprendizados ao longo de trajetórias pessoais e coletivas. Outra referência para o ensaio é o conceito de transição paradigmática baseada tanto na concepção inicial de Kuhn ^2^ como, posteriormente, nas contribuições de escolas pós-coloniais, em especial as Epistemologias do Sul e noções como justiça cognitiva e ecologia de saberes ^3^.

A ideia de transição paradigmática surgiu de debates epistemológicos nas últimas décadas em torno dos critérios de produção, legitimação e validação do conhecimento científico, impulsionados por dinâmicas que fortalecem a interdisciplinaridade. A crescente relevância de teorias abrangentes, como a de sistemas e da complexidade, tem sido marcantes no campo da Saúde Coletiva, incorporando autores como Ilya Prigogine, Isabelle Stengers e Edgar Morin, junto com a noção de determinação social dos processos de saúde e doença ^4^^,^^5^. Recentemente, contribuições críticas são formuladas pelas Ciências Sociais e Humanas a partir de abordagens pós-coloniais que articulam emancipação social, interdisciplinaridade e interculturalidade em torno das ideias de colonialidade do poder, do saber e do ser ^6^, com debates específicos, por exemplo, sobre possíveis conexões entre a Epidemiologia e a Antropologia em torno do conceito de cultura ^7^. Ou ainda sobre justiça cognitiva e ecologias de saberes ^3^ como alternativas para reconhecer e validar o diálogo entre conhecimentos produzidos dentro e fora da ciência moderna, dada a pluralidade interna e externa ao meio científico, de sistemas de conhecimentos que respondem às necessidades práticas, políticas, simbólicas e espirituais nas diferentes sociedades ^8^.

Neste ensaio, partimos da noção de paradigma de Kuhn ^2^, relacionado a consensos partilhados pelos membros de uma comunidade científica, num dado período, sobre pontos de vista acerca de problemas e possibilidades de soluções, influenciando o desenvolvimento de um conjunto de teorias, métodos e instrumentos que analisam, preveem e controlam certa realidade. Porém, toda teoria dentro de uma comunidade de pares sempre está submersa num paradigma dominante mais amplo, sutil e frequentemente não explicitado, vinculado a valores e dimensões sociais, humanas e filosóficas inerentes à sociedade e ao momento histórico no qual é produzido e usado. Todo paradigma reflete o espírito de uma época e é por isso que, ao falarmos de transição paradigmática, fugimos de uma posição positivista que enxerga a evolução da ciência “normal” ^9^, no sentido kuhniano, como algo restrito a uma comunidade especializada de pares. Uma posição positivista que defende os debates científicos restritos aos especialistas acaba por produzir uma tendência ao silenciamento ou não questionamento do paradigma hegemônico ante os desafios simultaneamente científicos e sociais de certa época. O tensionamento ao paradigma dominante é colocado em prática por uma comunidade ampliada de pares ^10^ que abarca novos sujeitos, sejam eles acadêmicos que incorporam novos referenciais pela inter/transdisciplinaridade, sejam eles provenientes de movimentos sociais, como trabalhadores, camponeses, indígenas, ambientalistas, ou atingidos pelo racismo ou machismo. Nesse sentido, a transição paradigmática corresponderia aos processos que desafiam paradigmas vigentes pela elaboração tanto de novos problemas como de soluções alternativas aos mesmos.

A principal base institucional, acadêmica e empírica do autor para construir o ensaio é a Saúde Coletiva brasileira nas áreas de saúde do trabalhador e saúde e ambiente. O trabalho se desenvolveu em duas dimensões coletivas: uma acadêmica, vinculada a pesquisas e pós-graduação predominantemente na Saúde Pública; e outra política e institucional, relacionada principalmente ao movimento sanitarista e à construção do SUS e seu sistema de vigilância (nas áreas de Saúde do Trabalhador e Ambiental) em sua busca inicial por democracia e justiças social e sanitária. A base empírica sobre os acidentes, portanto, esteve simultaneamente vinculada à estruturação da vigilância do SUS e às pesquisas, dissertações e teses que buscavam avançar, teórica e metodologicamente, na compreensão dos acidentes.

A liberdade do ambiente acadêmico propiciou que certos referenciais e mesmo articulações com movimentos sociais por vezes se afastassem da lógica de construção do SUS e do movimento sanitarista, como veremos mais à frente no que denominamos de terceiro movimento. Ao mesmo tempo que estudos acadêmicos incorporavam novos referenciais sobre a análise e prevenção de acidentes e desastres, eles serviam para apoiar práticas de investigação e vigilância de acidentes envolvendo redes frequentemente - mas nem sempre - protagonizadas pelo SUS em conjunto com universidades, sindicatos, movimentos sociais, ONGs ambientalistas e comunitárias, Ministérios Públicos e órgãos ambientais, entre outros.

A trajetória de experiências foi organizada em três movimentos marcados por contextos políticos, acadêmicos e institucionais distintos. Cada movimento apresenta um conjunto de experiências profissionais singulares articulado com a busca e a incorporação de escolas de pensamento que apresentavam novos referenciais teóricos e conceitos. Esses buscavam renovar a análise e a intervenção em torno dos acidentes e desastres, os quais tornaram-se objetos de investigação em função da mobilização de redes e coletivos diversos ao longo do tempo. A ideia de transição paradigmática adotada no artigo expressa os processos de desconstrução e reconstrução sobre a concepção de acidentes, suas origens, consequências e formas de prevenção que confrontavam paradigmas dominantes que não respondiam aos interesses de transformação apresentados pelos coletivos e ações sociais com os quais atuávamos em cada movimento.

Para cada um dos três movimentos, selecionamos alguns conceitos estratégicos utilizados para repensar os elementos da transição paradigmática em torno da análise e prevenção de acidentes e desastres a partir de diálogos interdisciplinares apoiados por certos autores e escolas teóricas, os quais continuam a representar, na avaliação do autor, contribuições relevantes para pensar desafios acadêmicos e de transformação social na atualidade.

## Primeiro movimento: prevenção simbólica e artificial na saúde dos trabalhadores

O contexto do primeiro momento está fortemente relacionado à construção da reforma sanitária e do SUS no processo de redemocratização da sociedade brasileira após a ditadura militar, civil e empresarial entre as décadas de 1980 e meados de 1990. A transição paradigmática nesse movimento buscou desconstruir formas de análise e prevenção de acidentes e desastres que culpabilizavam os trabalhadores pelos acidentes e ocultavam falhas organizacionais e tecnológicas dos processos de trabalho nas fábricas, ao mesmo tempo que negavam a importância dos saberes operários e de processos mais participativos para a prevenção.

Vários jovens pesquisadores e pesquisadoras se dedicaram à construção da área de Saúde do Trabalhador na Saúde Coletiva e de novas práticas de vigilância com forte participação de sindicatos e centrais sindicais envolvidos na redemocratização do país e com suas lutas por melhores condições de trabalho. Diálogos interdisciplinares com grupos profissionais e acadêmicos aproximaram profissionais do movimento sanitarista com as Ciências Sociais e, especialmente relevante na trajetória do autor, das engenharias, as quais são estratégicas para a análise dos processos de trabalho, suas tecnologias e riscos. Em termos empíricos, as ações de vigilância em saúde do trabalhador se deram a partir da criação dos conselhos estaduais e municipais criados com a participação de organizações sindicais, muitas vezes em parceria com os Ministérios Públicos. Os estados do Rio de Janeiro e São Paulo formaram um espaço privilegiado de atuação pela força de alguns setores econômicos, acadêmicos e movimentos sindicais, com a atuação marcante de profissionais militantes que transitavam entre a academia e o SUS, como Machado ^11^, Vasconcellos & Ribeiro ^12^ e Lacaz ^13^.

Um aspecto a ser ressaltado nesse primeiro movimento, expressão singular da trajetória do autor deste ensaio, são as contribuições provenientes do diálogo interdisciplinar com a pós-graduação do Instituto Alberto Luiz Coimbra de Pós-graduação e Pesquisa de Engenharia (Coppe) da Universidade Federal do Rio de Janeiro (UFRJ) na construção da saúde do trabalhador. A engenharia de produção estava sendo construída desde os anos 1970 como um dos suportes para a modernização industrial no país ao estudar e projetar sistemas de produção envolvendo o trabalho, seus ambientes, tecnologias e organizações. No Coppe/UFRJ, abriram-se frestas para a criação de um espaço libertário, permitindo a realização de trabalhos interdisciplinares e engajados. Iniciou-se uma forte interface com as ciências sociais e humanas na análise crítica dos processos de trabalho capitalistas ^14^, com influência em disciplinas como a ergonomia e a segurança e higiene do trabalho, as quais foram incorporadas na formação de quadros que iriam atuar nas ações de vigilância em saúde do trabalhador. Em especial, como forma de memória e tributo, destacamos três engenheiros interdisciplinares muito ativos desse período que já não estão mais entre nós: Miguel de Simoni partiu em 2002, Oswaldo Sevá em 2015, e Luiz Antonio Meirelles em 2017, todos com influência marcante em grupos da Saúde Coletiva, bem como na formação intelectual e existencial do autor. Os três trabalharam em parceria com médicos atuantes na construção da Saúde do Trabalhador à época, como Anamaria Tambellini (Fundação Oswaldo Cruz - Fiocruz) e Volney Câmara (UFRJ). O espírito interdisciplinar e libertário influenciado por esses e outros intelectuais da engenharia de produção permanece vivo nas - infelizmente poucas - organizações que continuam atuando em atividades que integram ativismo, tecnologia social, economia solidária, extensão, pesquisa e ensino. São exemplos o Núcleo de Solidariedade Técnica (Soltec/UFRJ), ligado ao Núcleo Interdisciplinar para Desenvolvimento Social (Nides/UFRJ), e a Rede de Engenharia Popular Oswaldo Sevá (Repos).

Um importante desafio acadêmico para a transição paradigmática desse primeiro momento foi desconstruir concepções de prevenção de acidentes atrasadas e ainda hegemônicas à época no meio empresarial e no Estado brasileiro. Por exemplo, dentro do Ministério do Trabalho, responsável pelas fiscalizações dos ambientes de trabalho até o advento do SUS, ainda predominavam abordagens ditas técnicas, porém ideológicas e reducionistas, que culpabilizavam os trabalhadores pelos acidentes apoiados na teoria de atos e condições inseguros ^15^.

As abordagens oriundas da saúde coletiva da época estavam mais preocupadas com a determinação social e a apropriação de conceitos propostos de origem sociológica marxista, como os importantes trabalhos de Laurell & Noriega ^16^ que relacionavam as formas de desgaste e cargas dos trabalhadores, expressos na geração de doenças e acidentes, com os processos de produção de mais valia absoluta e relativa. Outro clássico importante em termos epistemológicos e metodológicos, este mais pragmático, foi o livro *Ambiente de Trabalho: a Luta dos Trabalhadores pela Saúde* de Re et al. ^17^, publicado em 1986 e recentemente reeditado, que apresentou a origem da luta sindical italiana e influenciou a construção da ergologia no Brasil ^18^.

O diálogo interdisciplinar e engajado da engenharia de produção e da ergonomia da atividade de origem francesa com a saúde do trabalhador contribuiu para a transição paradigmática da análise e prevenção de acidentes e desastres com três conceitos importantes de prevenção: simbólica, artificial e real. O conceito de prevenção simbólica foi estratégico em toda nossa trajetória posterior, pois marcou a compreensão de que a transição paradigmática em sociedades com relações sociais e de trabalho pouco democráticas e autoritárias implicava processos de desocultamento da realidade estudada que obstaculizavam a transformação social. O conceito de prevenção simbólica foi apresentado pelo já citado Oswaldo Sevá e tem sua origem nos trabalhos do sociólogo francês Denis Duclos ^19^^,^^20^. Suas pesquisas entre os anos de 1970 e 1980 estavam voltadas para a análise da percepção dos riscos de trabalhadores da indústria química na França e nos Estados Unidos e demonstravam uma tendência ao apagamento ou à anulação das diferentes visões e experiências concretas, principalmente dos trabalhadores, acerca dos riscos e da prevenção de acidentes. Esse aparente consenso produzia uma forma de prevenção específica, denominada de simbólica, bem diferente do que poderia ser chamada de prevenção real efetivamente voltada ao controle de riscos e redução dos acidentes. Para Duclos ^19^^,^^20^, a prevenção simbólica tinha como principal função o controle da força de trabalho e da estabilização das relações sociais de produção dentro das fábricas, buscando convencer a todos de que os riscos estavam sob controle e as medidas preventivas eram efetivas. As percepções mais reais sobre as condições de trabalho eram invisibilizadas pela prevenção simbólica, porém subsistiam de diferentes maneiras nos trabalhadores e seus coletivos, e emergiam pela justaposição de discursos contraditórios sobre a confiança e a eficiência do controle de perigos existentes.

Tais posições críticas surgiam de forma mais evidente e explosiva em contextos das crises pós-acidentes, um fenômeno interpretado como um apelo à realidade do risco que desestabilizava a função social da prevenção simbólica, pelo menos até seu retorno pela circulação de discursos, relatórios e medidas punitivas das empresas e instituições que tentavam reestabilizar as relações sociais de trabalho e produção. Nesse contexto, ficava evidente a função não apenas simbólica e normativa, mas também controladora e opressora de teorias como a dos atos inseguros, que apresentavam os erros humanos como a principal causa dos acidentes. O outro lado da moeda era a possibilidade de rupturas e oportunidades para a transição paradigmática que surgiam nas frestas abertas pelas crises pós-acidentes, abrindo espaços para mobilizações e reconstruções de referenciais conceituais, políticas e ações em processos instituintes. A perspectiva emancipatória na época ocorreu pela incorporação dessa visão crítica nas ações de vigilância em saúde do trabalhador. Tais ações envolviam profissionais do SUS, engenheiros e outros especialistas em segurança e higiene do trabalho em conjunto com trabalhadores e seus sindicatos, frequentemente em contextos de denúncias de empresas e setores com acidentes graves.

A transição paradigmática almejada por essa visão crítica e pelas ações de vigilância visavam transformar a visão hegemônica à época sobre os acidentes e pode ser resumida da seguinte forma: em sociedades desiguais e autoritárias com forte concentração econômica e de poder, a análise e prevenção de acidentes e desastres fortalece o papel simbólico e artificial da prevenção de forma a normalizar os perigos das tecnologias e processos de trabalho, em vez de servir como motor efetivo de transformação pela melhoria das condições de trabalho e democratização da sociedade. Mais à frente, vários sociólogos corroboraram tal concepção com a ideia de anormalidade normal ^21^, para analisar os riscos tecnológicos e organizacionais ocultados, cujas manifestações iriam se expressar em acidentes num crescente de gravidade até produzirem eventos como mortes e desastres. Portanto, ao ocultar os riscos, a prevenção simbólica defendia os interesses capitalistas e suas gerências, assumindo, de forma não explícita, uma estratégia de controle da força de trabalho que se assumia como prevenção técnica, mas que correspondia a um simulacro da realidade do trabalho vivida no cotidiano.

Por sua vez, principalmente no contexto de desestabilização provocada pelos acidentes graves, surgiam descontentamentos, mobilizações e protagonismos dos trabalhadores e sindicatos organizados, movidos por revoltas e sede de justiça. Essa energia de mobilização foi aproveitada pelo SUS em construção, que fortaleceu parcerias entre movimentos sindicais e setores do aparelho de Estado num contexto de redemocratização da sociedade. As ações de vigilância e os relatórios produzidos, ancorados pelos novos referenciais e por vezes com o apoio do Ministério Público, pressionavam para desconstruir as narrativas oficiais das empresas e agências governamentais.

Às contribuições de Duclos devemos adicionar a importância da ergonomia de origem francesa voltada ao estudo do trabalho humano ^22^ ou ainda numa perspectiva mais interdisciplinar voltada à saúde, pela psicopatologia e psicodinâmica do trabalho de Dejours ^23^, ambas com forte presença na engenharia de produção e Saúde Coletiva daquele período. Uma importante descoberta da ergonomia francesa com implicações para a análise e prevenção de acidentes e desastres está relacionada aos conceitos de trabalho prescrito (tarefa) e trabalho real (atividade), bem como os conflitos e os problemas decorrentes das diferenças entre ambos. O trabalho prescrito está relacionado aos projetos das tecnologias, estações de trabalho e organizações, bem como às normas e exigências de produção (qualidade, produtividade e comportamento) definidas pela gerência. Já o trabalho real expressa como é realizado pelos trabalhadores numa atividade, incluindo os processos cognitivos envolvidos na avaliação e nas decisões do que é feito, pois eles cotidianamente precisam lidar com as exigências de produção impostas pela gerência e as variabilidades não previstas, além de defenderem suas vidas e serem afetados pelas diferentes cargas de trabalho, incluindo o cansaço, o estresse e vários tipos de sofrimento. Para a ergonomia sempre existem diferenças entre a tarefa prevista e a atividade realizada, e sociedades autoritárias e hierárquicas tendem a acentuar tais diferenças pela inexistência de interações e diálogos entre projetistas e gerências com os trabalhadores. Isso dificulta a humanização das condições de trabalho, agravado quando o próprio aparelho de estado e suas instituições são pouco democráticas e próximas ao empresariado, inviabilizando a proposta tripartite da Organização Internacional do Trabalho (OIT). Esse desequilíbrio é evidente em sociedades com fortes desigualdades e assimetrias sociais, fortalecendo o poder da gerência que defende a lógica do capital e da produtividade, enquanto os trabalhadores mais vulneráveis defendem suas vidas, sua dignidade, mas também seus empregos. Dessa forma, os trabalhadores não podem desenvolver e usar com mais plenitude diversas habilidades individuais e coletivas para levar a cabo seu trabalho de forma mais segura e humana, arriscando-se diante dos perigos e das contradições com as exigências da gerência, somadas à má qualidade dos projetos e da manutenção dos vários equipamentos e ambientes. A análise e prevenção de acidentes e desastres pode ser vista como resultante dessas contradições e conflitos, expressando o estágio dos atrasos ou das conquistas na direção de sociedades mais justas, democráticas e inclusivas.

Por isso, a transição paradigmática implicava, naquele momento, em introduzir dispositivos que acessassem o trabalho real e tornassem evidente a prevenção simbólica e o gerenciamento artificial dos riscos, conceito desenvolvido por autores como Machado ^11^. Para desconstruir a prevenção artificial, era estratégico adotar e difundir metodologias participativas envolvendo os trabalhadores e seus coletivos dentro de um processo mais amplo de democratização da sociedade, viabilizado pela criação de instâncias participativas com sindicatos de trabalhadores nas ações de vigilância. Tais ações apoiaram-se na determinação social e no modelo operário italiano ^17^ para orientar a vigilância em saúde do trabalhador pelo SUS em construção à época. Portanto, o objetivo da transição paradigmática desse momento era apontar medidas tecnológicas e organizacionais mais democráticas e participativas que valorizassem a vida dos trabalhadores.

## Segundo momento: a complexidade dos desastres ambientais, vulnerabilidades e a proposta da ciência pós-normal para lidar com as incertezas

O contexto político-institucional desse movimento apresenta um quadro paradoxal: apesar da maior liberdade para a organização de movimentos sindicais e outros da sociedade civil no período pós-ditadura, inclusive do próprio movimento sanitarista em torno da implementação do SUS, a emergência de governos e políticas neoliberais geraram restrições aos avanços das ações de vigilância em saúde do trabalhador. Um fato marcante em meados dos anos 1990 para a análise e prevenção de acidentes e desastres foi a passagem dos acidentes de trabalho para os acidentes e desastres ambientais, que nas indústrias químicas foram caracterizados como acidentes químicos ampliados nos trabalhos do cientista social Carlos Machado de Freitas junto com o autor, em diálogo com movimentos sindicais. Ele e Jorge Machado foram os principais companheiros dessa jornada nos anos 1990, e, em 2000, organizamos um livro ^24^ sobre o tema que, entre outras propostas, apresentava a Análise Interdisciplinar e Participativa de Acidentes.

Um evento emblemático da época foi a ocorrência de um acidente na fábrica química da multinacional alemã Bayer pouco dias antes da Conferência das Nações Unidas sobre Meio Ambiente e Desenvolvimento (CNUMAD ou Rio-92) no Rio de Janeiro em 1992, quando uma nuvem tóxica foi liberada e atingiu Belford Roxo, localizado na Baixada Fluminense, periferia do Rio de Janeiro. Apesar da empresa e o órgão ambiental estadual divulgarem nota que afirmava não haver perigo, diversos moradores apresentaram sintomas de intoxicação química, muitos sendo levados às pressas para o atendimento de urgência em postos de saúde e hospitais próximos. Na época, várias ações de vigilância foram feitas envolvendo o sindicato dos trabalhadores, o SUS, o Ministério Público do Trabalho, universidades e a Fiocruz. A primeira dissertação de mestrado orientada pelo autor no Programa de Pós-graduação em Saúde Pública (PPGSP), defendida em 1995 ^25^, teve por objeto esse acidente. Desde então, vários projetos e orientações ligados tanto ao PPGSP da Escola Nacional de Saúde Pública Sergio Arouca (ENSP) como outros programas de pós-graduação envolveram questões relacionadas à análise e prevenção de acidentes e desastres.

Esse evento tornou evidente que, ao romper os muros fabris, os desastres ambientais representavam um fenômeno bem mais complexo que exigia novas abordagens. Os primeiros estudos bibliográficos e cooperações com instituições internacionais mostraram um vasto conjunto de publicações sobre o tema com a visibilidade dos chamados, à época, acidentes maiores (*major accidents*), como os ocorridos na cidade de Seveso na Itália, em 1976, e outro na cidade de Bhopal na Índia em 1984. Este último envolveu a liberação de um gás altamente tóxico (isocianato de metila) numa fábrica multinacional dos Estados Unidos (Union Carbide) que produzia agrotóxicos em cooperação com o governo indiano para viabilizar a “revolução verde” naquele país. O desastre de Bhopal é considerado o pior acidente industrial da história em termos de mortes no curto (cerca de 3 mil) e médio prazos (mais de 20 mil), sendo emblemático para países como o Brasil e outros com industrialização tardia.

A aproximação com dois referenciais teóricos foi muito importante nesse período para compreender a complexidade desses eventos e avançar na transição paradigmática sobre a análise e prevenção de acidentes e desastres em diálogo, crescente desde então, com a comunidade acadêmica internacional. O primeiro é o conceito de vulnerabilidade social, que já vinha sendo trabalhado na saúde pública ^26^. Em nosso caso, nos aproximamos do conceito de vulnerabilidade construído no campo dos desastres ^27^, que, a partir das ciências sociais, buscava responder a seguinte questão: como explicar o fato de que desastres ditos “naturais” ou tecnológicos de magnitude semelhantes, em termos da liberação de energias ou substâncias químicas perigosas, poderiam gerar consequências tão radicalmente distintas em termos de mortes? Não casualmente, esse diferencial de mortes era mais gritante na comparação entre os países mais ricos da Europa Ocidental, América do Norte ou Japão, com países como Brasil, México e Índia. Esse fenômeno já era discutido anteriormente por autores conhecidos, como Barry Castleman ^28^ e seu conceito de duplo padrão. Seu objetivo foi analisar como a proibição de tecnologias e substâncias perigosas, caso do cancerígeno amianto, ocorria nos países mais ricos e “desenvolvidos” enquanto, num caminho inverso, era cada vez maior a importação, produção e consumo nos países “em desenvolvimento”, como o Brasil.

Nossa entrada no debate acadêmico internacional sobre o tema teve por marco a publicação de um artigo ^29^ em 1996 na *Risk Analysis*, prestigiada revista internacional sobre análise e gerenciamento de riscos. A intenção foi introduzir o debate geopolítico e da divisão internacional do trabalho e dos riscos usando por referência o sociólogo Immanuel Wallerstein sobre o sistema/mundo capitalista ^30^. Num longo processo de discussão com os pareceristas tivemos que retirar do trabalho termos como países periféricos e semiperiféricos, considerados “ideológicos”, para chegar a um acordo que colocou em seu lugar “países em industrialização”, ao invés de “em desenvolvimento”, conceito proposto pelos revisores e terminantemente recusado pelos autores. Esse exemplo tornou evidente que a transição paradigmática seria complexa no plano acadêmico internacional, já que o debate científico mais “objetivo e neutro” e “menos ideológico” era controlado por centros de pesquisa dos países mais poderosos, chamados de “desenvolvidos”, os países centrais do sistema/mundo de Wallerstein ^30^, subordinando artigos e financiamentos de projetos de pesquisa aos cânones hegemônicos de qualidade.

Outro referencial de especial relevância que apresentou a dimensão epistemológica para o nosso debate foi a ciência pós-normal, cujo contato inicial ocorreu casualmente em 1993 com a visita do autor ao Joint Research Centre localizado em Ispra, Itália, no contexto do doutorado sanduíche que realizava na Europa. A partir dessa época, foram iniciados diálogos de grande profundidade epistemológica e metodológica junto aos dois principais autores dessa proposta, Funtowicz & Ravetz ^10^^,^^31^. Em síntese, a proposta da ciência pós-normal resulta de um contexto político, econômico e institucional da Europa Ocidental preocupada em como lidar, de forma democrática, com uma sociedade crescentemente complexa envolvendo múltiplos riscos, a então chamada sociedade do conhecimento, da informação e do risco. A ciência pós-normal propunha como alternativa uma nova forma de gestão de problemas complexos, principalmente ambientais, relacionados à interface ciência-política ao assumir que a ciência especializada ou normal desconsiderava incertezas, o peso dos valores nas tomadas de decisão e a pluralidade de perspectivas legítimas. O aumento das incertezas demandaria um modo de gestão mais democrático e participativo, já que problemas como contaminações químicas, nucleares ou biológicas não poderiam ser resolvidos apenas por especialistas e era necessária uma comunidade ampliada de pares.

Ainda que sua difusão no campo da saúde coletiva brasileira tenha se circunscrito a um grupo acadêmico restrito, destacamos algumas contribuições da ciência pós-normal. Primeiro, a importância do refinamento epistemológico da qualidade do conhecimento para avaliar e comunicar os três tipos de incertezas na ciência pós-normal: de natureza técnica, como a imprecisão dos instrumentos de medição e seus usos; metodológica, relativa ao domínio de teorias de produção e análise de dados; e epistemológica, relacionada à carência de teorias mais consistentes para lidar com a complexidade do fenômeno. Enquanto as duas primeiras poderiam ser superadas por avanços na aquisição e na manutenção de equipamentos e treinamento científico de profissionais em distintos níveis, que expressaria o grau de desenvolvimento técnico-científico e tecnológico de um país ou região, a última refletiria o mais elevado grau de incerteza: a ignorância epistêmica. Em seu limite, poderia ser irredutível quanto mais complexo e misterioso fosse o problema em questão, o que levaria a ciência a assumir uma perspectiva mais humilde e sábia ao reconhecer o que não se sabe. Para a ciência pós-normal, a ciência normal, no sentido dado por Kuhn ^2^, tendia a afirmar mais certezas que explicitar suas incertezas, as quais permaneciam ocultas. Isso dificultaria a transição paradigmática ao facilitar a manipulação da produção científica de acordo com os interesses que financiavam ou definiam as condições do trabalho científico e profissional especializado, principalmente no contexto de processos decisórios sobre o que seriam riscos aceitáveis ou não.

A solução para esse dilema passaria tanto pela capacidade de explicar as incertezas em jogo, como pela educação e popularização científica no conjunto da sociedade, que apresenta condições legítimas de participar dos processos decisórios, e não apenas os especialistas. A utopia da nova sociedade da informação caminharia para um processo contínuo de aperfeiçoamento e qualificação do diálogo em ambientes de debates abertos, democráticos e robustos que evitariam conflitos sociais mais radicais pela polarização de posições e interesses.

A construção de comunidades ampliadas de pares proposta pela ciência pós-normal pode ser considerada uma utopia razoavelmente ingênua por desconsiderar os conflitos sociais e de interesse presentes em sociedades capitalistas desiguais e com fortes assimetrias de poder. Sua proposta, para funcionar, dependeria de um elevado nível moral, de sinceridade, inteligência e disposição ao diálogo por parte dos vários grupos envolvidos na tomada de decisões complexas. Esse seria o caso, por exemplo, da liberação de tecnologias perigosas como as nucleares, químicas e biológicas, ou ainda os agrotóxicos e transgênicos necessários ao modelo do agronegócio. Mas como esperar esse elevado nível moral, de robustez argumentativa, de abertura para o diálogo e sabedoria por parte de políticos, empresários e grupos religiosos quando se tornam muito poderosos e dogmáticos? Os exemplos bem atuais relacionados às mudanças climáticas e às ameaças à democracia com a expansão da extrema direita e das *fake news* mostram o fracasso da utopia europeia da sociedade democrática da informação.

Ao mesmo tempo, a evolução do *modus operandi* científico nos apresenta um paradoxo de difícil superação para as áreas mais sofisticadas e especializadas da ciência: como cientistas e técnicos formados ao longo de muitos anos, que aderiram a certos paradigmas e adotaram uma gramática particular de difícil compreensão aos não especialistas, a exemplo da física nuclear, da economia e da epidemiologia, irão se abrir para cruzar as fronteiras que podem contradizer, expor fragilidades e colocar em questão os valores, pressupostos e certezas dos paradigmas e modelos que operam? Na realidade, encontrávamos um fechamento por parte de comunidades especializadas típicas do funcionamento da ciência normal e de processos decisórios tecnoburocráticos.

Esse problema tornou evidente o papel estratégico de especialistas alternativos, contra ou anti-hegemônicos, para a produção de análises e relatórios de contra-*expertise* que pudessem reconhecer e traduzir os limites das teorias e métodos mais sofisticados para a análise e prevenção de acidentes e desastres. Eles funcionam como uma espécie de “anjos da guarda” técnico-científicos engajados que atuam na desestabilização de posições hegemônicas técnicas ao explicitarem os pressupostos, bifurcações metodológicas, incertezas, interesses e diferentes resultados em jogo, por vezes radicalmente diferentes dos apresentados nas mídias hegemônicas. Elas ocultavam sistematicamente incertezas e interesses em jogo apresentados pelos relatórios oficiais de empresas, de instituições governamentais ou mesmo acadêmicas, porém com conflitos de interesse dissimulados.

A prevenção simbólica da análise e prevenção de acidentes e desastres, apontada no momento anterior, persistia nos relatórios de licenciamento ambiental ou sobre desastres envolvendo tecnologias e processos produtivos complexos e perigosos. Porém, nesse segundo movimento, os efeitos iam além dos espaços fabris, já que os perigos se ampliavam em escalas espaciais, temporais e sociais bem mais abrangentes. Ou seja, tornou-se evidente o papel da prevenção simbólica em estabilizar as relações sociais de produção e o paradigma produtivista ou neoliberal do progresso científico e tecnológico que subjazia ante o processo de globalização em curso. O segundo movimento serviu de passagem para um novo momento de transição paradigmática em que se acentuavam os desastres, tanto fabris como ecológicos, mais amplos, enquanto novas lutas por emancipação social surgiam no país e no planeta.

##  O momento atual: lutas por justiças ambiental e cognitiva, e a prevenção abissal *versus* emancipatória para enfrentar desafios contemporâneos 

Para encerrar, apresentamos o terceiro e atual momento com a proposta de prevenção abissal e prevenção emancipatória que completa nossa trajetória conceitual e empírica na virada para o século XXI até os tempos atuais. É de especial interesse para a atualidade, pois reflete um contexto contraditório, paradoxal e de forte complexidade pelo agravamento de inúmeras crises, no plano global, regional, nacional e local, tanto socioambientais e sanitárias como crises econômicas, existenciais e ontológicas. Essas últimas estão relacionadas ao questionamento sobre o sentido moderno de progresso e de humanidade, o que nos exige pensar cada vez mais a noção de emancipação social a partir da necessária conexão entre transição paradigmática e outra mais ampla, de natureza civilizatória em uma sociedade cada vez mais globalizada.

No plano nacional, viveu-se, desde 2003, uma era de governos federais do Partido dos Trabalhadores (PT) com políticas redistributivas e participativas favorecidas pelo crescimento econômico. A incorporação no novo governo de várias lideranças sindicais e políticas atuantes reforçou a institucionalização de políticas públicas e ações do Estado voltadas à equidade, redução da pobreza, combate à fome, participação e inclusão social. Ao mesmo tempo, intensificou um paradoxo entre ciclos instituintes e instituídos envolvidos na transformação social. A ideia de engajamento social e de transição paradigmática depende do fortalecimento de processos instituintes contra-hegemônicos ou, como preferimos, anti-hegemônicos, que contribuem para alterar o *status quo* que mantém desigualdades e injustiças. Contudo, o desequilíbrio entre processos instituintes e instituídos em democracias fracas torna-se mais evidente quando conquistas sociais são instáveis e rapidamente revertidas. No Brasil, essa fragilidade foi exposta pelas limitações do governo de coalizão do PT envolvendo grupos de centro-direita, o qual foi encerrado tragicamente em 2016 com o impeachment da presidenta Dilma Rousseff e a ascensão, em 2018, de um governo de extrema-direita, o que parecia impensável até então. Essa é uma das explicações para que, nesse terceiro movimento, o engajamento social relacionado à análise e prevenção de acidentes e desastres acabasse se aproximando de redes e organizações relativamente independentes do Governo Federal e das instituições, caso da Rede Brasileira de Justiça Ambiental (RBJA).

Com relação ao plano internacional, a globalização e o neoliberalismo caminhavam a passos largos com o fim da União das Repúblicas Socialistas Soviéticas (URSS), revertendo os aparentes avanços trilhados com a Rio-92 em torno do problemático conceito de desenvolvimento sustentável, atrelando a prática da democracia à ideia de governança. O quadro global de crise democrática mostra como a própria ideia de democracia forte é colocada em xeque nas regiões mais consideradas estáveis e resilientes, como países da Europa Ocidental e da América Norte. Paralelamente às políticas de inclusão, reforçou-se o desenvolvimentismo, o consumismo e o neoextrativismo em países com maior potencial de produção de *commodities* agrícolas e minerais intensificadas pela globalização. Houve uma expansão e aceleração da precarização do trabalho e da degradação ecológica, da reprimarização da economia, bem como da flexibilização neoliberal das regras sociais, ambientais, trabalhistas e previdenciárias. No Brasil, isso impulsionou ainda mais diferentes formas de espoliação e violência nos territórios, principalmente os tradicionais (como indígenas e quilombolas) e da agricultura familiar, para a expansão de projetos do agronegócio, mineração, logísticas e infraestruturas associadas. Dialeticamente, esse processo foi acompanhado por resistências cada vez mais organizadas por parte de povos e comunidades camponesas, indígenas e quilombolas, além de populações das periferias urbanas. Nesse contexto, tornou-se cada vez mais importante se aproximar e compreender o significado dessas resistências e lutas sociais protagonizadas por tais sujeitos, já que processos oficiais instituídos que conformavam políticas públicas e práticas institucionais, ainda que bastante resilientes para não desmoronarem rapidamente, como o SUS, foram se afastando de utopias e processos emancipatórios em curso de transformação social.

Para compreender esse quadro complexo, utilizamos referenciais teóricos de dois campos interdisciplinares com forte engajamento social. O primeiro é o da ecologia política e os movimentos por justiça ambiental ^32^, e o segundo, das abordagens pós-coloniais, entre elas as epistemologias do Sul, as quais privilegiam a análise de lutas sociais por reconhecimento de outras formas de ser, poder e saber ^3^^,^^6^. Aproximam, portanto, a política entendida em seu sentido clássico com as políticas territoriais, ontológicas e do conhecimento, e acrescentam duas novas dimensões de justiças (ambiental e cognitiva) às duas anteriormente privilegiadas pela Saúde Coletiva (social e sanitária) ^33^. Esses referenciais causaram contribuições inovadoras para compreender temas candentes que vinham se intensificando com a crescente centralidade de questões ecológicas e ontológicas, incluindo o ambientalismo, o racismo e o feminismo. Houve uma atualização da geografia política já presente na construção da Saúde Coletiva pela ideia do retorno do território como expressão de disputas entre o global e o local, ou o vertical e o horizontal nos termos de Santos ^34^.

À dimensão socioecológica, agregaram-se as contribuições das abordagens pós-coloniais para ampliar a leitura geopolítica do sistema-mundo capitalista e de fenômenos como o racismo e as diversas formas de violência e opressão contra povos e comunidades originários, de matriz africana, mulheres, entre outras. Com isso, houve um deslocamento conceitual dos países capitalistas dependentes ou do Terceiro Mundo, que passaram a fazer parte de um grupo mais amplo denominado de Sul Global. Mais que uma definição geográfica de países (existe um Norte dentro do Sul Global, assim como um Sul dentro do Norte Global), trata-se de um conceito que expressa as heranças e consequências do colonialismo (ou colonialidade) ^3^. Introduz uma política ontológica (definições do humano) que critica estratégias de dominação e invisibilização dos povos do Sul Global pelo Norte Global, metáfora da modernidade ocidental hegemônica.

A justiça cognitiva, proposta pelas epistemologias do Sul, propõe compreender e apoiar os processos emancipatórios que buscam reconhecer e legitimar outras formas de ser, saber e viver em sociedade e com a natureza presentes no Sul Global, as quais foram - e continuam a ser - desprezadas ou aniquiladas pela combinação dos três eixos de dominação moderna: o capitalismo, o colonialismo (ou colonialidade) e o patriarcado. Portanto, para além da permanência de formas de exclusão social decorrentes da exploração capital-trabalho, propõe-se a exclusão radical de natureza ontológica, tal como denominada por Santos ^(^^3^^)^ e Santos & Meneses ^35^, expressa pela ideia de pensamento abissal que marca a modernidade eurocêntrica. As exclusões radicais envolvem questões como o racismo e a própria ciência moderna, quando impõe cânones que desprezam e não dialogam com sistemas de conhecimento tradicionais e populares do Sul Global (epistemicídios), como os dos povos originários, de matriz africana e camponeses.

As reflexões para a transição paradigmática sobre a análise e prevenção de acidentes e desastres vêm sendo construídas nesse momento pela crescente relevância de desastres e tragédias que o autor atuou produzindo relatórios contra hegemônicos e manifestações públicas em conjunto com diversos movimentos, organizações e entidades como a RBJA, a Associação Brasileira de Saúde Coletiva (Abrasco) e a Articulação Nacional de Agroecologia (ANA). Num primeiro momento com indústrias químicas e de petróleo, como o vazamento de óleo na Baía de Guanabara (Rio de Janeiro, 2000), o naufrágio da plataforma P-36 na Bacia de Campos (Rio de Janeiro, 2001), o vazamento da barragem em Cataguases (Minas Gerais, 2003) e, mais recentemente, o derrame de óleo que atingiu o litoral do Nordeste em 2019. Em 2011, ocorreu o desastre climático com as fortes chuvas e deslizamentos na região serrana que matou cerca de mil pessoas. Por fim, as tragédias da megamineração do aço, como o rompimento da barragem de Mariana (Minas Gerais), em 2015 na empresa Samarco, e outro rompimento de barragem em Brumadinho (Minas Gerais, 2019) da empresa Vale, com 270 mortes ^36^. Isso sem contar a tragédia ambiental do agronegócio químico-dependente que produz incêndios, desmatamentos e contaminação em massa pelo uso intensivo de agrotóxicos indispensáveis aos monocultivos de grande extensão ^37^.

O paradigma emergente na investigação sobre esses desastres destaca que territórios camponeses, indígenas, quilombolas e de pescadores tradicionais são fortemente invisibilizados e atingidos pelos projetos de desenvolvimento econômico do modelo capitalista neoextrativista. Nesse novo momento, passamos a relacionar as causas sistêmicas desses desastres tanto com o modelo de desenvolvimento como com processos de invisibilização, de racismo ambiental e de epistemicídios presentes que marcam tanto os processos de licenciamento ambiental como as análise e prevenção de acidentes e desastres posteriores aos eventos. Dessa forma, a prevenção simbólica se alia a uma prevenção abissal que mantém ilesos o modelo de desenvolvimento defendido pelos governos em várias instâncias e as empresas responsáveis, além de gerar os desastres em nome do progresso e do crescimento econômico.

Para enfrentar a prevenção abissal que invisibiliza grupos sociais e territórios atingidos e suas lutas em função de sua classe social, raça e gênero, propomos a ideia de uma prevenção emancipatória. Ela busca a transição paradigmática da análise e prevenção de acidentes e desastres em convergência com a transição civilizatória ao reconhecer a existência e dialogar respeitosamente com os diversos saberes e práticas dos vários povos tradicionais que atuam como guardiões da natureza e de seus territórios sagrados, e constroem propostas como o Bem Viver e Conviver. Opõem-se à perspectiva exploradora, controladora e não convivencial da modernidade ocidental e sua concepção utilitarista de progresso. Dessa forma, o desastre transforma-se, mais que um evento de magnitude quantificável, numa metáfora de nossa crise civilizatória, e a esperança de sua compreensão pode guiar o desafio da transição paradigmática em várias áreas que podem convergir para a reinvenção da emancipação social em nossos tempos atuais.

## Conclusões

Embora o foco do ensaio esteja nos acidentes e desastres, acreditamos que diferentes áreas e objetos de investigação poderão se inspirar nas reflexões aqui apresentadas para pensarem potencialidades de transição paradigmática e emancipação social junto aos campos, áreas de conhecimento e mesmo disciplinas especializadas que atuam, desde que abertas a abordagens interdisciplinares e socialmente engajadas. Os desafios das transições paradigmática e civilizatória tornam-se, com o agravamento das crises ecológica e democrática, exigências cada vez mais transversais ao conjunto da academia.

Nas discussões apresentadas sobre análise e prevenção de acidentes e desastres, num primeiro momento, o papel da prevenção simbólica era estabilizar relações sociais de trabalho ocultando as contradições da exploração capitalista e práticas organizacionais autoritárias e, como alternativa, propunha-se uma prevenção que considerasse o trabalho real e a participação dos trabalhadores. Num segundo momento, a prevenção simbólica persistia, porém seu entendimento ampliou-se para além do mundo fabril. Para manter estáveis as relações sociais de produção diante de uma sociedade globalizada em crise socioambiental crescente, era necessário ocultar incertezas e interesses ante processos de trabalho, tecnologias e riscos cada vez mais complexos e incertos. A transição paradigmática do segundo movimento propunha processos decisórios mais democráticos em comunidades ampliadas de pares, incluindo territórios e populações vulnerabilizados em áreas de risco junto com ambientalistas, além de contra especialistas que pudessem traduzir e desconstruir as argumentações ditas “técnicas” acerca das tecnologias controladas com riscos aceitáveis. Por fim, no terceiro momento, agudizam-se crises socioambientais, sanitárias, do modelo de desenvolvimento, do Estado e da própria ciência, apresentando uma crise simultaneamente paradigmática e civilizatória. Os desastres surgem imbricados com questões de racismo, das exclusões radicais promovidas pela noção de progresso, controle e pretensa superioridade ontológica da modernidade eurocêntrica. Global e local interconectam-se cada vez mais, como na ideia de “glocal”, e a crise socioecológica impulsionou o reconhecimento de uma nova era: a do antropoceno, ou capitaloceno.

Acreditamos que as respostas a esses desafios passam, do ponto de vista da produção de conhecimentos, por novas epistemologias que promovam encontros respeitosos entre saberes da academia com os sistemas tradicionais e populares de conhecimento e sabedoria dos povos do Sul Global, sejam eles originários das Américas, África e Ásia, os quais se atualizam permanentemente em novos hibridismos que conformam novos sistemas de saberes e práticas. As agendas ligadas aos movimentos ecologistas, de povos e comunidades tradicionais, antirracistas e feministas, apresentam tendências tanto emancipatórias como potencialmente fragmentadoras quando suas diversas lutas se opõem umas às outras. Articular diversidade e convivencialidade na produção de conhecimentos com qualidade é um desafio estratégico para a academia em tempos de tanta belicosidade e *fake news*.

Nesse sentido, o novo desafio do conhecimento transcende o proposto há mais de 30 anos por Minayo ^38^ para pensar a pesquisa em saúde, pois implica em reconhecer e dialogar com sistemas de saberes e produzir conhecimentos interdisciplinares e interculturais junto com os movimentos sociais e os territórios, de forma a apoiar simultaneamente movimentos de transição paradigmática e processos emancipatórios que lutam por transformação social num mundo que vive uma crise planetária.

O novo desafio do conhecimento reaproxima a questão epistemológica da transformação social e uma nova compreensão do sentido de emancipação. Para isso, precisaremos de mais consciência, filosofia, arte e engajamento social para a construção de uma ciência sensível e aberta às exigências dos novos tempos.

## References

[1] Jara OH (2018). La sistematización de experiencias, práctica y teoría para otros mundos
posibles.

[2] Kuhn TS (1997). A estrutura das revoluções científicas.

[3] Santos BS (2018). O fim do império cognitivo.

[4] Paim JS, Almeida N (1998). Saúde coletiva uma "nova saúde pública" ou campo aberto a novos
paradigmas?. Rev Saúde Pública.

[5] Almeida ND (1997). Transdisciplinaridade e saúde coletiva. Ciênc Saúde Colet.

[6] Mignolo WD, Walsh CE (2018). On decoloniality: concepts, analytics, praxis.

[7] Sevalho G (2022). Contribuições das críticas pós-colonial e decolonial para a
contextualização do conceito de cultura na Epidemiologia. Cad Saúde Pública.

[8] Santos BS, Meneses MPG, Nunes JA, Santos BS (2004). Semear outras soluções: os caminhos da biodiversidade e dos
conhecimentos rivais.

[9] Minayo MC (2010). Disciplinaridade, interdisciplinaridade e
complexidade. Emancipação.

[10] Funtowicz S, Ravetz J (1994). Uncertainty, complexity and post-normal science. Environ Toxicol Chemistry.

[11] Machado JMH (1997). Processo de vigilância em saúde do trabalhador. Cad Saúde Pública.

[12] Vasconcellos LCF, Ribeiro FSN (1997). Investigação epidemiológica e intervenção sanitária em Saúde do
Trabalhador. Cad Saúde Pública.

[13] Lacaz FADC (2007). O campo Saúde do Trabalhador resgatando conhecimentos e práticas
sobre as relações trabalho-saúde. Cad Saúde Pública.

[14] Braverman H (1987). Trabalho e capital monopolista.

[15] Jackson JM, Vilela RAG, Garcia EG, Almeida IM (2013). Sobre a "aceitabilidade social" dos acidentes do trabalho e o
inaceitável conceito de ato inseguro. Rev Bras Saúde Ocup.

[16] Laurell AC, Noriega M (1989). Processo de produção e saúde: trabalho e desgaste operário.

[17] Re A, Marri G, Briante G, Oddone I, Chiatella M, Glória S (2020). Ambiente de trabalho: a luta dos trabalhadores pela saúde.

[18] Ramminger T, Athayde MRC, Brito J (2013). Ampliando o diálogo entre trabalhadores e profissionais de
pesquisa alguns métodos de pesquisa-intervenção para o campo da saúde do
trabalhador. Ciênc Saúde Colet.

[19] Duclos D, Fabiani J-L, Theys J (1987). La société vulnérable - évaluer et maîtriser les risques..

[20] Duclos D (1989). Le peur et les savoirs: la societé face à la science, la technique et
leurs dangers.

[21] Wynne B (1988). Unruly technology practical rules, impractical discourses and
public understanding. Soc Stud Sci.

[22] Wisner A (1987). Por dentro do trabalho: ergonomia, método e técnica.

[23] Dejours CH (1987). A loucura do trabalho: estudo de psicopatologia do trabalho.

[24] Freitas CM, Porto MFS, Machado JMH (2000). Acidentes industriais ampliados: desafios e perspectivas para o controle
e a prevenção.

[25] Vasconcellos ES (1995). O atendimento médico de emergência nos acidentes químicos ampliados. Rio
de Janeiro.

[26] Ayres JR (1997). Sobre o risco: para compreender a epidemiologia.

[27] Cutter SL (1996). Vulnerability to environmental hazards. Prog Hum Geogr.

[28] Castleman B (1983). The double standard in industrial hazards. Int J Health Serv.

[29] Porto MF, Freitas CM (1996). Major chemical accidents in industrializing countries: the
sociopolitical amplification of risk.. Risk Anal.

[30] Wallerstein I (1979). The capitalist world-economy.

[31] Funtowicz S, Ravetz J (1993). Science for the post-normal age. Futures.

[32] Alimonda H (2015). Ecología política latinoamericana y pensamiento crítico:
vanguardias arraigadas. Desenvolvimento e Meio Ambiente.

[33] Porto M F, Rocha DF, Fasanello MT (2021). Saúde, ecologias e emancipação: conhecimentos alternativos em tempos de
crise(s).

[34] Santos M (2005). O retorno do território. OSAL - Observatório Social da América Latina.

[35] Santos BS, Meneses MP (2014). Epistemologias do Sul.

[36] Porto MFS (2019). A tragédia da mineração e do desenvolvimento no Brasil desafios
para a saúde coletiva. Cad Saúde Pública.

[37] Carneiro FF, Augusto LGS, Rigotto RM, Friedrich K, Búrigo AC (2015). Dossiê ABRASCO: um alerta sobre os impactos dos agrotóxicos na
saúde.

[38] Minayo MCS (2006). O desafio do conhecimento: pesquisa qualitativa em saúde..

